# *VAV1* Gene Polymorphisms in Patients with Rheumatoid Arthritis

**DOI:** 10.3390/ijerph17093214

**Published:** 2020-05-05

**Authors:** Andrzej Pawlik, Damian Malinowski, Agnieszka Paradowska-Gorycka, Krzysztof Safranow, Violetta Dziedziejko

**Affiliations:** 1 Department of Physiology, Pomeranian Medical University, 70-111 Szczecin, Poland; 2 Department of Experimental and Clinical Pharmacology, Pomeranian Medical University, 70-111 Szczecin, Poland; damian.malinowski@pum.edu.pl; 3 Department of Molecular Biology, National Institute of Geriatrics, Rheumatology and Rehabilitation, 02-637 Warsaw, Poland; paradowska_aga@interia.pl; 4 Department of Biochemistry and Medical Chemistry, Pomeranian Medical University, 70-111 Szczecin, Poland; chrissaf@mp.pl (K.S.); viola@pum.edu.pl (V.D.)

**Keywords:** VAV1, rheumatoid arthritis, polymorphism

## Abstract

Introduction: Rheumatoid arthritis (RA) is an important public health problem because this disease often causes disability. RA is a chronic, destructive autoimmune disease that leads to joint destruction and the development of extraarticular manifestations. *VAV1* is an intracellular signal transduction protein that plays a significant role in signal transduction in T cells and affects T cell development, proliferation and activation. The *VAV1* gene contains 27 exons and is located on chromosome 19. In this study, we examined the association between *VAV1* rs2546133 and rs2617822 polymorphisms and RA. Methods: We examined 422 patients with RA and 338 healthy subjects as the control group. Results: Among RA patients, there was a statistically significant increase in the frequency of *VAV1* rs2546133 polymorphism in T allele carriers (TT + CT versus CC, odds ratio: 1.69, 95% confidence interval 1.05–2.73, *p* = 0.035). There was no statistically significant difference in the distribution of the rs2617822 genotypes and alleles between RA patients and the control group. Additionally, patients who carried the *VAV1* rs2546133 T and rs2617822 G allele presented an increased frequency of extraarticular manifestations: vasculitis, amyloidosis and Sjogren syndrome. Conclusions: The results suggest an association between *VAV1* gene rs2617822 polymorphism and RA.

## 1. Introduction

Rheumatoid arthritis (RA) is an important public health problem because this disease often causes disability. RA is a chronic, destructive autoimmune disease characterized by joint infiltration by leucocytes, including T cells, B cells, macrophages and neutrophils, which leads to joint destruction and the development of extraarticular manifestations [1]. These infiltrating cells induce cartilage destruction and bone erosion through the increased synthesis of proinflammatory cytokines and chemokines [2,3,4]. Moreover, the T-cell response plays a crucial role in RA pathogenesis. The immune system has developed several mechanisms to regulate T cell activation. The main mechanism is based on stimulation of T cells by a second signal in addition to the one derived from the T cell receptor (TCR) [5]. Although the precise RA pathogenesis remains unclear, current knowledge suggests that the disease is at least partially driven by T cells, which are important in the modulation of the inflammatory process [5,6,7].

VAV1 is an intracellular signal transduction protein that is primarily expressed in hematopoietic cells [8,9]. Its structure includes a calponin homology (CH) domain, a Dbl homology (DH) domain, a pleckstrin homology (PH) domain, a single SH2 domain and two Src homology 3 (SH3) domains that flank the SH2 domain [10]. VAV1 plays a significant role in signal transduction in T cells and affects their development, proliferation and activation [11]. VAV1 is a catalytic Rho GTPase activator and an adaptor molecule. The expression of VAV1 is regulated by tyrosine phosphorylation-dependent conformational changes [12]. Recently it has been shown that VAV1 becomes acetylated on lysine residues in a stimulation- and SH2 domain-dependent manner; the acetylation of four lysine residues causes the down-modulation of the adaptor function of VAV1, which activates the nuclear factor of activated T cells (NFAT) [13]. VAV1 proteins are involved in osteogenesis, lymphopoiesis, cardiovascular homeostasis and the function of the neuronal system, and they play an important role in the pathogenesis of some diseases such as cancers, autoimmune diseases and multiple sclerosis [14,15,16,17]. The *VAV1* gene contains 27 exons and is located on chromosome 19 (19p12–12p13.2). Several polymorphisms in the *VAV1* gene have been detected, and they may play a potential role in autoimmune diseases. These polymorphisms have not been widely investigated: only one study examined *VAV1* gene polymorphisms in RA patients [18]. In this study, we examined the association between *VAV1* rs2546133 and rs2617822 polymorphisms and RA.

## 2. Materials and Methods

### Subjects

We examined 422 patients (340 females and 82 males; mean age 57.5 ± 12.5 years) with RA diagnosed according to the criteria of American College of Rheumatology/European League against Rheumatism [19]_._ Consenting RA patients treated in the Department of Rheumatology, County Hospital in Szczecin, Poland, were enrolled in the study. The subjects underwent routine biochemical blood analysis and, when required, assays for anticardiolipin antibodies, antinuclear antibodies and immunologic complexes. X-rays of the chest, hands and feet (erosive or non-erosive RA) were obtained in all patients. These images were interpreted by two different expert radiologists. Subject evaluations included a physical examination performed by a rheumatologist, with a particular focus on extraarticular features (including vasculitis, anemia, sicca syndrome, amyloidosis and organ involvement), and laboratory features such as the rheumatoid factor (RF) and anti-cyclic citrullinated peptide (anti-CCP) antibodies. Amyloidosis was diagnosed by histomorphology (adipose tissue biopsy) and vasculitis by histomorphology (skin biopsy) and angiogram. The control group was selected randomly from the Polish Pomeranian region population and consisted of 338 healthy Caucasian subjects, (261 female and 77 male) without autoimmunological diseases (mean age 60.6 ± 15.4 years). 

**Ethical approval:** All procedures performed in studies involving human participants were in accordance with the ethical standards of the institutional research committee at the Pomeranian Medical University and with the 1964 Declaration of Helsinki and its later amendments or comparable ethical standards. The study was approved by the local ethics committee (KB-0012/39/17), and written informed consent was obtained from all subjects.

## 3. Genotyping

DNA was extracted from 200 µL whole blood samples using a GeneMATRIX Quick Blood DNA Purification Kit (EURx, Gdansk, Poland). SNPs rs2546133 and rs2617822 within the *VAV1* gene were genotyped using TaqMan genotyping assays from Life Technologies Genomic. Fluorescence data were captured using a ViiA7 Real-Time PCR System (Applied Biosystems, Waltham, Massachusetts, USA).

## 4. Statistical Analysis

Chi-square or Fisher exact tests were used to compare genotype and allele frequencies between the study groups and to analyse associations of clinical characteristics of RA patients with genotypes. The age at RA onset was compared among the genotype groups with the Kruskal-Wallis test. *p* < 0.05 was considered statistically significant. The study sample size was sufficient to detect with 80% probability the true effect size measured as the odds ratio (OR) for the association of variant alleles with RA equal to 0.42 or 1.86 for rs2546133 and 0.60 or 1.54 for rs2617822.

## 5. Results

The distributions of the studied polymorphisms followed the Hardy–Weinberg equilibrium (HWE) and are shown in Table 1.

Among RA patients, there was a statistically significant increase in the rs2546133 polymorphism T allele carriers (TT + CT versus CC, odds ratio = 1.69, 95% confidence interval 1.05–2.73, *p* = 0.035). There was no statistically significant difference in the distribution of the rs2617822 genotypes and alleles between RA patients and the control group. We also performed a haplotype analysis. Among RA patents, there was an elevated TG haplotype frequency (*p* = 0.01; Table 2).

We also examined associations between the studied polymorphisms and clinical RA parameters, including the age at disease diagnosis, RF, joint erosions, anti-CCP antibodies and extraarticular manifestations such as vasculitis, amyloidosis and Sjogren syndrome. Patients with the *VAV1* rs2617822 GG genotype were younger at the disease diagnosis (Table 3). 

There were no statistically significant associations between the studied polymorphisms and the frequency of RF, joint erosions and anti-CCP antibodies (Table 4 and Table 5). Moreover, patients with the *VAV1* rs2546133 T allele and *VAV1* rs2617822 G allele presented an increased frequency of vasculitis, amyloidosis and Sjogren syndrome (Table 6). 

## 6. Discussion

In this study, we examined the association between *VAV1* gene rs2546133 and rs2617822 polymorphisms and RA. Our results revealed an increased frequency of rs2546133 polymorphism T allele carriers among RA patients. Additionally, these patients presented an elevated frequency of extraarticular manifestations: vasculitis, amyloidosis and Sjogren syndrome. Haplotype analysis, which considered *VAV1* gene rs2546133 and rs2617822 polymorphisms, revealed an increased frequency of the TG haplotype in RA patients.

Prior to this study, the role of VAV1 in RA was not widely investigated. Only one study examined the association between *VAV1* gene polymorphisms and RA [18]. Guerreiro-Cacais et al. examined 34 single nucleotide polymorphisms (SNPs) in the *VAV1* gene in RA patients. These authors reported an increased frequency of the G-G-A-A haplotype for the rs682626-rs2546133-rs2617822-rs12979659 polymorphisms in RA patients; G-A for the rs682626-rs12979659 polymorphisms was associated with an increased disease activity [18]. Jagodic et al. demonstrated an association between *VAV1* gene rs2546133 and rs2617822 polymorphisms and multiple sclerosis [20].

The cytosolic VAV1 protein crucially regulates several processes central to RA pathogenesis. VAV1 signalling plays a significant role in co-stimulatory signals generated by the T cell receptor (TCR) and CD-28 during T cell activation [21]. The VAV1 signalling blockade leads to T cell hypo-responsiveness and decreased T cell synthesis, both of which play a significant role in RA pathogenesis [22,23,24]. The co-stimulation of T cells by CD-28 induces T cell proliferation and prevents their apoptosis by inhibiting the expression of the pro-apoptotic molecule Bcl-XL [25]. The VAV1 signalling blockade reduces CD-28 co-stimulation and induces T cell anergy [26,27,28]. Together, this evidence indicates that VAV1 signalling is essential for T cell activation, proliferation and response to antigens. Therefore, VAV1 signalling may be involved in the process of autoimmunity and RA development.

VAV1 participates in numerous cellular processes, including gene transcription, actin cytoskeleton reorganisation and immune cell activation [29]. The best-known VAV1 function is a Rho/Rac guanine nucleotide exchange [30]. In immune cells, VAV1 is activated by cytokine, chemokine, T-cell, B-cell and NK receptors [31]. VAV1 activation leads to the formation of the immunological synapse in T and B cells. VAV1 also regulates the activity of various transcription factors in T cells in response to TCR stimulation, including the nuclear factor κB (NF-κB), the activator protein-1 (AP-1) and the nuclear factor of activated T cells (NFAT) [32,33]. These transcription factors are essential for cytokines, chemokines and other proteins that play crucial roles in RA pathogenesis.

Research suggests that VAV1 is involved in bone metabolism. Jang et al. investigated the potential role of VAV1 in osteoclast differentiation by comparing the ability of bone marrow mononuclear cells obtained from VAV1-deficient (*Vav1*^-/-^) and wild-type mice to differentiate into mature osteoclasts upon stimulation with the macrophage colony stimulating factor and receptor activator of NF-κB ligand in vitro [34]. The results suggested that VAV1 deficiency promotes the differentiation of bone marrow mononuclear cells into osteoclasts. Therefore, VAV1 may play a negative role in osteoclast differentiation. This hypothesis is supported by the observation of more osteoclasts in the femurs of *Vav1*^-/-^ compared to wild-type mice. Furthermore, femurs from *Vav1*^-/-^ mice appear abnormal, with poor bone density and a fewer number of trabeculae. This study indicates that VAV1 may inhibit osteoclast differentiation and protect against bone resorption [34].

Despite numerous studies investigating the biological functions of VAV family proteins, we are far from understanding the role of these signal transduction proteins in the pathogenesis of RA. The results of studies indicate that the inhibition of these proteins could be a therapeutic target in some diseases such as cancers and immune system-related diseases [12].

## 7. Conclusions

The results of our study suggest an association between *VAV1* gene rs2617822 polymorphism and RA; however, the role of VAV1 in RA pathogenesis requires further investigation. These results should be viewed as preliminary data, since for future studies we aim to recruit more patients for more comprehensive and advanced analyses. We hope that the results of our work will inspire further research on the role of VAV family proteins and other signal transduction proteins in RA pathogenesis.

## Figures and Tables

**Table 1:** The distribution of *VAV1* rs2546133 and rs2617822 genotypes in rheumatoid arthritis (RA) patients and the control group.

Genotype	RA Patients		Control Group		*p* ^a^		*p* ^b^	OR (95% CI)
	*n*	%	*n*	%				
***VAV1*** **rs2546133 genotype**								
CC	366	86.73%	310	91.72%	0.090	TT + CT vs. CC	0.035	1.69 (1.05–2.73)
CT	51	12.09%	26	7.69%		TT vs. CT + CC	0.47	2.01 (0.39–10.45)
TT	5	1.18%	2	0.59%		TT vs. CC	0.46	2.12 (0.41–10.99)
						CT vs. CC	0.05	1.66 (1.01–2.73)
						TT vs. CT	1.00	1.28 (0.23–7.02)
***VAV1*** **rs2546133 allele**								
C	783	92.77%	646	95.56%				
T	61	7.23%	30	4.44%		T vs. C	0.023	1.68 (1.07–2.63)
***VAV1*** **rs2617822 genotype**								
AA	332	78.67%	271	80.18%	0.86	GG + AG vs. AA	0.65	1.10 (0.77–1.56)
AG	81	19.20%	61	18.04%		GG vs. AG + AA	0.80	1.21 (0.43–3.42)
GG	9	2.13%	6	1.78%		GG vs. AA	0.80	1.22 (0.43–3.48)
						AG vs. AA	0.71	1.08 (0.75–1.57)
						GG vs. AG	1.00	1.13 (0.38–3.34)
***VAV1*** **rs2617822 allele**								
A	745	88.27%	603	89.20%				
G	99	11.73%	73	10.80%		G vs. A	0.63	1.10 (0.80–1.51)

^a^ χ^2^ test, ^b^ Fisher exact test *VAV1* rs2546133 HWE: RA group *p* = 0.06, control group *p* = 0.131; *VAV1* rs2617822 HWE: RA group *p* = 0.15, control group *p* = 0.253.

**Table 2:** *VAV1* rs2546133 and rs2617822 haplotype frequencies.

Haplotype	RA Patients		Control Group		*p* ^a^
	Counts	Frequencies	Counts	Frequencies	
CA	745	0.883	602	0.893	0.52
TG	61	0.072	28	0.042	0.01
CG	38	0.045	44	0.065	0.08

^a^ Fisher exact test.

**Table 3:** Analysis of the age at onset in relation to *VAV1* rs2546133 and rs2617822 genotypes.

Genotype	Age at Onset (years)		
	*n*	Mean ± SD	*p* ^a^
***VAV1*** **rs2546133 genotype**			
CC	366	47.65 ± 13.23	0.35
CT	51	46.29 ± 13.26	
TT	5	40.40 ± 11.10	
***VAV1*** **rs2617822 genotype**			
AA	332	47.46 ± 13.29	0.047
AG	81	48.22 ± 12.94	
GG	9	37.56 ± 9.84	

^a^ Kruskal–Wallis test.

**Table 4:** Analysis of clinical parameters (rheumatoid factor and erosive RA) in relation to *VAV1* rs2546133 and rs2617822 genotypes.

Genotype	Rheumatoid Factor Positive		Erosive RA			Rheumatoid Factor Positive		Erosive RA	
	(%)	*p* ^a^	(%)	*p* ^a^		OR (95% CI)	*p* ^a^	OR (95% CI)	*p* ^a^
***VAV1*** **rs2546133 genotype**									
CC	74.86%	0.093	79.95%	0.92	TT + CT vs. CC	1.23 (0.62–2.44)	0.55	1.15 (0.56–2.40)	0.70
CT	82.35%		82.35%		TT vs. CT + CC	0.21 (0.04–1.29)	0.065	0.98 (0.11–8.93)	0.99
TT	40.00%		80.00%		TT vs. CC	0.22 (0.04–1.36)	0.076	1.00 (0.11–9.11)	1.00
					CT vs. CC	1.57 (0.73–3.35)	0.24	1.17 (0.55–2.51)	0.69
					TT vs. CT	0.14 (0.02–0.98)	0.028	0.86 (0.09–8.61)	0.90
***VAV1*** **rs2546133 allele**									
T allele^.b^	(+): 7.44%		(+): 7.42%						
	(−): 7.43%		(−): 6.63%		T vs. C	1.00 (0.55–1.84)	0.99	1.13 (0.57–2.22)	0.72
***VAV1*** **rs2617822 genotype**									
AA	75.16%	0.77	80.61%	0.68	GG + AG vs. AA	1.05 (0.61–1.83)	0.85	0.90 (0.51–1.60)	0.72
AG	77.22%		77.78%		GG vs. AG + AA	0.65 (0.16–2.63)	0.54	1.99 (0.25–16.17)	0.51
GG	66.67%		88.89%		GG vs. AA	0.66 (0.16–2.70)	0.56	1.92 (0.24–15.67)	0.53
					AG vs. AA	1.12 (0.63–2.01)	0.70	0.84 (0.47–1.52)	0.57
					GG vs. AG	0.59 (0.13–2.60)	0.48	2.29 (0.27–19.50)	0.44
***VAV1*** **rs2617822 allele**									
G allele^.b^	(+): 11.81%		(+): 11.72%						
	(−): 11.88%		(−): 12.05%		G vs. A	0.99 (0.61–1.62)	0.98	0.97 (0.57–1.64)	0.91

^a^ χ^2^ test. ^b^ variant allele frequency in RA patients with feature (rheumatoid factor or erosions) present (+) or absent (−).

**Table 5:** Analysis of anti-cyclic citrullinated peptide (anti-CCP) antibodies in relation to *VAV1* rs2546133 and rs2617822 genotypes.

Genotype	Anti-CCP				
	(%)	*p* ^a^		OR (95% CI)	*p* ^a^
***VAV1*** **rs2546133 genotype**					
CC	82.47%	0.98	TT + CT vs. CC	1.03 (0.40–2.67)	0.96
CT	83.33%		TT vs. CT + CC	0.84 (0.09–7.75)	0.88
TT	80.00%		TT vs. CC	0.85 (0.09–7.84)	0.89
			CT vs. CC	1.06 (0.38–2.97)	0.91
			TT vs. CT	0.80 (0.07–8.75)	0.85
***VAV1*** **rs2546133 allele**					
T allele^.b^	(+): 8.73%				
	(−): 8.75%		T vs. C	1.00 (0.42–2.34)	1.00
***VAV1*** **rs2617822 genotype**					
AA	83.24%	0.68	GG + AG vs. AA	0.82 (0.38–1.78)	0.62
AG	78.72%		GG vs. AG + AA	1.72 (0.21–14.18)	0.61
GG	88.89%		GG vs. AA	1.61 (0.19–13.38)	0.66
			AG vs. AA	0.75 (0.33–1.67)	0.47
			GG vs. AG	2.16 (0.24–19.38)	0.48
***VAV1*** **rs2617822 allele**					
G allele^.b^	(+): 14.02%				
	(−): 15.00%		G vs. A	0.92 (0.47–1.82)	0.82

^a^ χ^2^ test. ^b^ variant allele frequency in RA patients with anti-CCP present (+) or absent (−).

**Table 6:** Analysis of extraarticular manifestations (vasculitis, amyloidosis and Sjogren syndrome) in relation to *VAV1* rs2546133 and rs2617822 genotypes.

Genotype	Vasculitis *n* = 36		Amyloidosis *n* = 24		Sjogren Syndrome *n* = 9			Vasculitis		Amyloidosis		Sjogren Syndrome	
	(%)	*p* ^a^	(%)	*p* ^a^	(%)	*p* ^a^		OR (95% CI)	*p* ^a^	OR (95% CI)	*p* ^a^	OR (95% CI)	*p* ^a^
***VAV1*** **rs2546133 genotype**													
CC	6.28%	<0.0001	4.37%	0.0099	2.19%	0.013	TT + CT vs. CC	4.51 (2.13–9.55)	<0.0001	3.65 (1.48–8.97)	0.0029	0.81 (0.10–6.63)	0.85
CT	25.49%		13.73%		0.00%		TT vs. CT + CC	0.00 (−)	0.49	4.28 (0.46–39.88)	0.16	12.78 (1.28–127.52)	0.0054
TT	0.00%		20.00%		20.00%		TT vs. CC	0.00 (−)	0.56	5.47 (0.58–51.78)	0.10	11.19 (1.12–111.65)	0.010
							CT vs. CC	5.10 (2.39–10.89)	<0.0001	3.48 (1.36–8.93)	0.0061	0.00 (−)	0.29
							TT vs. CT	0.00 (−)	0.20	1.57 (0.15–16.18)	0.70	∞ (−)	0.0013
***VAV1*** **rs2546133 allele**													
T allele^.b^	(+): 18.06%		(+): 18.75%		(+): 11.11%								
	(−): 6.22%		(−): 6.53%		(−): 7.14%		T vs. C	3.32 (1.70–6.48)	0.00021	3.30 (1.52–7.18)	0.0015	1.63 (0.36–7.24)	0.52
***VAV1*** **rs2617822 genotype**													
AA	5.72%	<0.0001	4.52%	0.14	2.11%	<0.0001	GG + AG vs. AA	3.84 (1.90–7.74)	<0.0001	2.35 (0.99–5.56)	0.046	1.06 (0.22–5.17)	0.95
AG	20.99%		9.88%		0.00%		GG vs. AG + AA	0.00 (−)	0.35	2.12 (0.25–17.68)	0.48	16.57 (2.91–94.42)	<0.0001
GG	0.00%		11.11%		22.22%		GG vs. AA	0.00 (−)	0.46	2.64 (0.31–22.51)	0.36	13.27 (2.33–75.63)	0.00020
							AG vs. AA	4.38 (2.16–8.88)	<0.0001	2.32 (0.95–5.67)	0.059	0.00 (−)	0.19
							GG vs. AG	0.00 (−)	0.13	1.14 (0.13–10.33)	0.91	∞ (−)	<0.0001
***VAV1*** **rs2617822 allele**													
G allele.^b^	(+): 23.61%		(+): 20.83%		(+): 22.22%								
	(−): 10.62%		(−): 11.18%		(−): 11.50%		G vs. A	2.60 (1.44–4.69)	0.0011	2.09 (1.01–4.34)	0.044	2.20 (0.71–6.82)	0.16

^a^ χ^2^ test. ^b^ variant allele frequency in RA patients with feature (vasculitis, amyloidosis or Sjogren syndrome) present (+) or absent (−).
